# Potential adverse actions of prenatal exposure of acetaminophen to offspring

**DOI:** 10.3389/fphar.2023.1094435

**Published:** 2023-04-05

**Authors:** Ka Wu, Wensheng Lu, Xin Yan

**Affiliations:** ^1^ Department of Pharmacy, The Second People’s Hospital of Nanning City, The Third Affiliated Hospital of Guangxi Medical University, Nanning, China; ^2^ Department of Endocrinology, The Second People’s Hospital of Nanning City, The Third Affiliated Hospital ofGuangxi Medical University, Nanning, China

**Keywords:** prenatal exposure, acetaminophen, offspring, disruption effect, hepatotoxicity

## Abstract

Acetaminophen (APAP) is a widely used as analgesic and antipyretic drug. APAP is also added as an active ingredient in various medications to relieve pain and reduce fever. APAP has been widely used in pregnant women in the past decades because it is considered a relatively safe drug with recommended dose in different countries. However, an increasing number of epidemiological and experimental studies have shown that APAP exposure during pregnancy may increase the risk of inducing reproductive and neurobehavior dysfunctions, hepatotoxicity in offspring. This review aims to assess the potential effects of prenatal APAP exposure on offspring growth and development.

## 1 The effects and characteristics of APAP used in pregnancy

APAP is a common antipyretic that has been used widely in family population, including pregnant woman (Nilsen et al., 2023). As a household remedy, pregnant women through self-directed or unintentional use of APAP is unavoidable (Bandoli et al., 2020). It is estimated that parts of pregnant women may expose to APAP in gestation period (Patel et al., 2022). It is reported that pregnant women usually take over-the-counter drugs (including APAP) without medical guidance and it may adversely affect the fetus development (Thiele et al., 2013). Experimental data *in vivo* show neonatal APAP exposure (single low dose with 30 mg/kg) may induce adverse actions on the developing brain (Philippot et al., 2022). Prenatal APAP exposure may induce autism spectrum disorders in childhood, suggesting that gestational APAP use should be clinically guided (Ji et al., 2020). Pharmacokinetic analysis of oral APAP dose (single intake 1,000 mg) shows that the contents are highly correlated in maternal venous blood (12.3 μg/mL) and fetal blood (11.2 μg/mL) (Nitsche et al., 2017). It is reported that APAP content in umbilical cord blood is approximate 3.6 mg/L as the median molar dose proportion metabolized to acetaminophen-sulphate and N-acetyl-p-benzoquinone imine is 0.8% and 0.06% (Mian et al., 2020). Compared to non-pregnant women, an increase in volume of distribution and an increase in clearance of APAP in pregnant women is 3.5%–60.7% and 36.8%–84.4% respectively. Notably, the toxic metabolite N-acetyl-p-benzoquinone imine is greatest in the first trimester, followed by the second and third trimester in pregnant women (Brookhuis et al., 2021). During pregnancy and breastfeeding periods, woman may use APAP to relieve acute or chronic and it may cause negative consequence to offspring (Scialli et al., 2010). Maternal APAP use during pregnancy is implicated in certain adverse outcomes in offspring, including attention and sleep problems (Sznajder et al., 2022). The cohort studies report that prenatal APAP exposure results in adverse neurodevelopment, including attention-deficit/hyperactivity disorder associated with frontoparietal network brain connectivity (Ystrom et al., 2017). Relief from maternal pain is an important factor for smooth delivery, and it is particularly important to use drugs rationally according to the type and duration of symptoms (Roberge et al., 2016). However, for various reasons, they are reported to take at least one or two medications, both prescription and over-the-counter, during pregnancy (Lupattelli et al., 2014). APAP is commonly used as antipyretic and analgesic, including pregnant women (Bauer et al., 2021). Despite the controversy occurs, APAP has potential benefits in treating clinical symptoms in pregnant women and it is considered to be low-risk (Toda, 2017). Collectively, current evidences partially reason that prenatal exposure of APAP may affect the development and function in offspring.

## 2 APAP-exerted neuroendocrine disrupting actions

**FIGURE 1:**
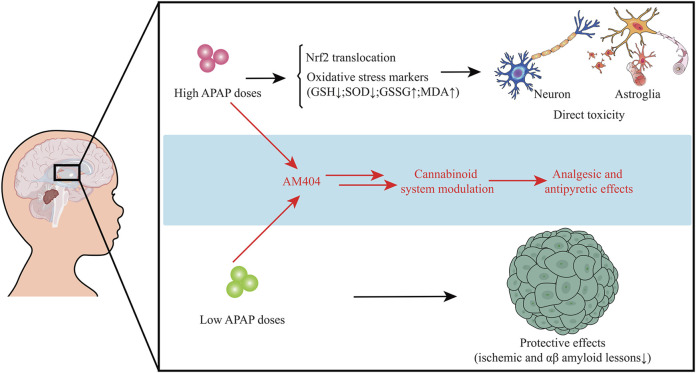
High dose of APAP may induce oxidative stress and affect Nrf2 function resulting in reduction of oxidative stability and increment of toxic response to cause direct toxicity to neuron and astroglia. However, low dose of APAP may mediate neuroprotective actions. Low and high APAP doses can exert analgesic and antipyretic actions through regulation of cannabinoid system.

Mounting data suggest that APAP may exert neuroendocrine disrupting effects, in which APAP exposure may affect endocrinological functions from fetal life to adulthood. Increasing reports manifest that APAP is a hormone disrupter that interferes with sex and thyroid hormones required for normal brain development (Aminoshariae and Khan, 2015). Exposure of APAP during a key period of brain development can lead to a long-term effect on cognitive functions (Viberg et al., 2014). As APAP can pass through the blood-brain barrier, it can act both centrally and peripherally through different molecular regulatory mechanisms. For example, one of the mechanisms of APAP action is thought to relieve pain is through competitive suppression of the peroxidase moiety of prostaglandin H2 synthase and regulation of cannabinoid receptor signaling pathway (Bührer et al., 2021). Additionally, APAP has also been found to inhibit serotonergic mechanisms for relieving pain in clinical studies (Pickering et al., 2008). However, the oxidative brain impairment and mitochondrial dysfunction are found in APAP acute exposure *in vivo* under 600 mg/kg for 5 h (da Silva et al., 2012). Collectively, understanding the exact mechanisms regarding APAP-caused neuroendocrinological dysfunction may be indefinable. However, the potential disrupting effects of APAP seem to be closely related to dosing dependence. High dose of APAP exposure may induce oxidative stress and affect Nrf2 function resulting in reduction of oxidative stability and increment of toxic response to cause direct toxicity to neuron and astroglia. Instead, low dose of APAP exposure may mediate neuroprotective actions *via* reducing ischemic and amyloid impairment. Both low and high APAP doses can play analgesic and antipyretic effects *via* modulation of cannabinoid system (Figure 1).

## 3 APAP-affected reproductive functions

**FIGURE 2:**
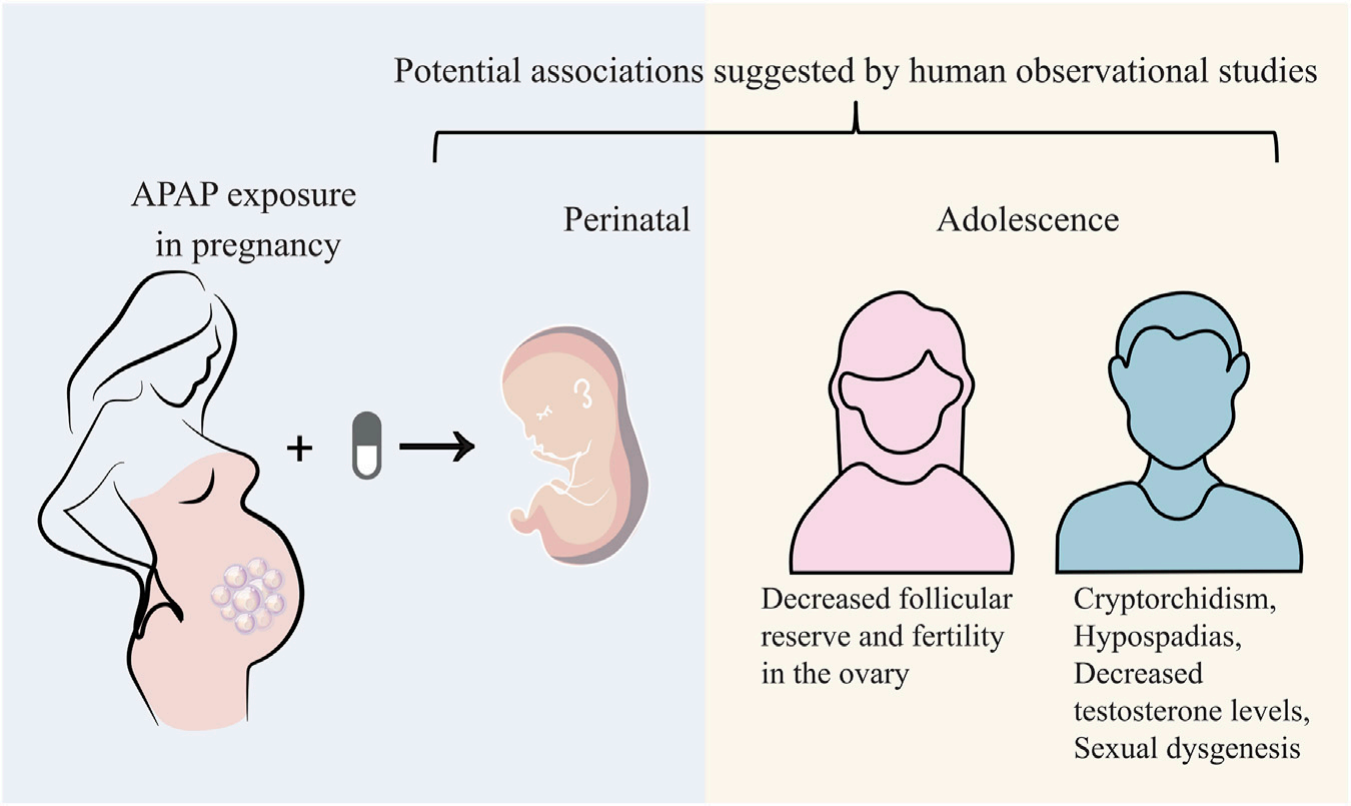
Prenatal APAP exposure may potentially disrupt human reproductive functions both male and female offspring.

Exposure of APAP *in utero* is involved in an increased risk of developing male genital tract abnormalities. An *in vitro* study from isolating adult human testis suggests that 10^−4^–10^−5^ mol/L APAP exposure for 24–48 h may alter testosterone and insulin-like factor 3 in Leydig cells (Albert et al., 2013). Another *ex vivo* study from isolating human ovarian fragments displays that 10^−3^–10^−8^ mol/L APAP exposure for 7 days may decrease the total cell number ovaries and the KI67-positive cell density, induce cell death. Human fetal ovarian steroidogenesis is affected (Lecante et al., 2022). A Danish National Birth Cohort study investigating 47,400 live-born singleton sons reports that maternal exposure of APAP over 4 weeks during pregnancy, especially within the first and second trimesters, may potentially elevate the presence of cryptorchidism in offspring (Henriksen and Olsen et al., 2010). A prospective birth cohort study involving 2,500 pregnant women shows that exposure to analgesics including APAP during pregnancy is related to a reduced anogenital distance (AGD) in offspring boys that may affect normal reproductive development (Lind et al., 2017). A prospective study from 2,229 recruited women in UK exhibits that intrauterine exposure of APAP during 8–14 weeks (masculinisation programming window) of gestation is correlated to shortened AGD from birth to 24 months (Fisher et al., 2016). A longitudinal Puberty Cohort study in Danish demonstrates that APAP use during pregnancy and postpartum may induce advance appearance of female pubertal development around 1.5–3 months earlier (Ernst et al., 2019). In addition to experimental data, there are still few human studies on the long-term reproductive functions affected by prenatal APAP exposure, especially for intergenerational and transgenerational outcomes. In brief, current evidences partially reason that prenatal exposure of APAP may potentially disrupt human reproductive functions both male and female offspring (Figure 2).

## 4 APAP-impacted human neurobehavior functions

Increasing evidences data indicate APAP use during pregnancy may induce the potential risk of developing neurobehavior problems and hyperkinetic symptoms (Liew et al., 2014). Another An Auckland Birthweight Collaborative study from 871 infants shows that APAP exposure during pregnancy may increase the risk of developing attention-deficit/hyperactivity disorder (ADHD)-like symptoms in children at 7 and 11 years of age (Thompson et al., 2014). Prenatal and postnatal exposure of APAP up to 18 months result in autism spectrum conditions (ASC) and ADHD in offspring (Alemany et al., 2021). A prospective birth cohort from 7,796 mothers presents that APAP use in 18–32 weeks of pregnant women results in increased risk of multiple behavioral problems, including emotional symptom (Stergiakouli et al., 2016). The meta-analysis systematic review, meta-analysis, and meta-regression analysis of cohort studies from 132,738 mother-child pairs suggests that prenatal APAP use is involve in the elevated for inducing ADHD, autism spectrum disorder (ASD) and hyperactivity syndromes (Masarwa et al., 2018). Eight cohort studies included 244,940 participants exhibits that APAP exposure to pregnancy may raise the risk of ADHD in offspring, in which a longer duration of prenatal APAP exposure is likely associated with a higher risk outcome (Gou et al., 2019). The available data is of observational nature only. However, current demographical data is of observational nature only, and pathological mechanism regarding APAP-induced human neurobehavior dysfunction is limitedly revealed in details.

## 5 APAP-induced hepatoxic effects

**FIGURE 3:**
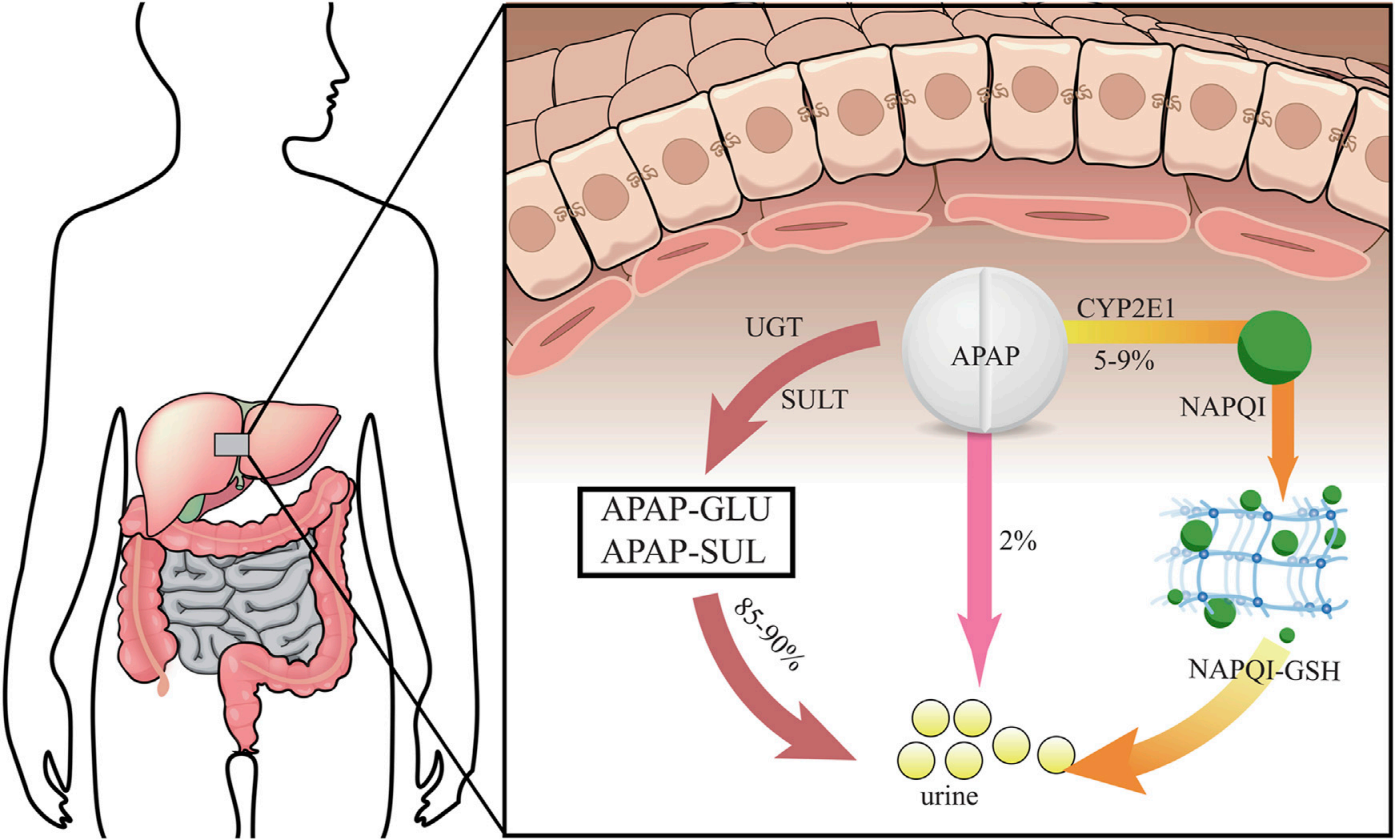
Human APAP is metabolized and detoxified in liver tissue *via* different processes before urine excretion.

**FIGURE 4:**
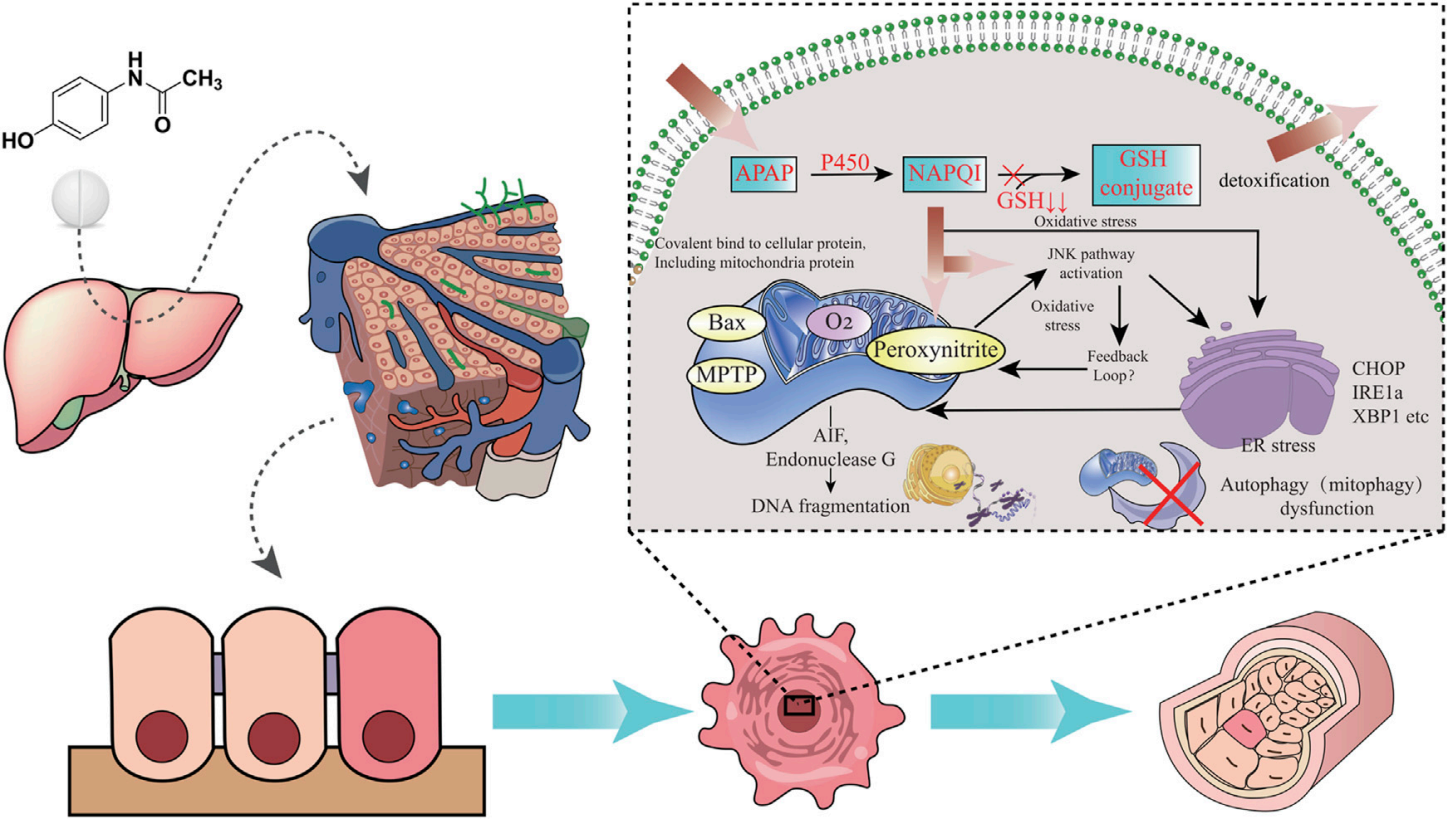
Molecular mechanism of APAP-induced hepatotoxicity is revealed through integrated pathways, including oxidative stress and ER stress.

Usually, most of patients take around 12 g or more APAP before inducing serious hepatotoxicity, and the peak serum transaminase activity can occur between 48 and 96 h. It may cause liver failure in APAP-used patients after days (Fisher et al., 2016). It is preclinically showed that prenatal APAP exposure may induce hepatic toxicity in offspring owing to oxidative stress and inflammatory injury (Rofaeil et al., 2023). Other preclinical evidences indicate that an increased susceptibility towards APAP-induced liver injury in pregnant mice, and hematopoietic stem cells in fetal liver is functionally affected (Karimi et al., 2015). The liver is the largest detoxifying organ in human body. When APAP enters the body, it will undergo “first pass” metabolism in the liver tissue approximately 25% of APAP before being excreted in the urine as glucuronide and sulphate conjugates (Prescott, 1980). Physiologically, most of APAP is metabolized by stage II binding enzymes characterized as UDP-glucuronate transferase (UGT) and sulfonyltransferase (SULT) for further being converted into non-toxic compounds. Another part of APAP can react with cytochrome P450 enzymes (CYP) and is eventually metabolized to the highly reactive intermediate metabolite N-acetyl-p-benzoquinoneimide (NAPQI), a strong hepatotoxic molecule (Lancaster et al., 2015). It is reported that exposure to NAPQI metabolized by APAP may lead to hepatotoxicity and acute liver failure, in which this outcome may affect the fetus and newborn developments (Brune et al., 2015). Typically, NAPQI is rapidly detoxified by glutathione (GSH) action. However, APAP overdose use can cause functional insufficiency of specific metabolic enzymes owing to threshold saturation, resulting in NAPQI exhausting GSH (James et al., 2003). APAP in human body is metabolized and detoxified in liver tissue, as reveled in a graphical briefing (Figure 3). Mitochondrial oxidative stress is one of the leading causes in APAP-induced liver damage. Additionally, certain cellular events including autophagy, endoplasmic reticulum stress, inflammatory infiltration and microcirculatory dysfunction, have been found involvement with the pathogenesis of APAP-caused liver injury (Yan et al., 2018). An animal study shows that prenatal exposure to APAP may reduce the expressions of insulin receptor substrate 1 (IRS1), phosphorylated glycogen synthase kinase-3beta (GSK-3β) and protein kinase B (AKT), and downregulate hepatic glucose transporter 2 (GLUT2) in offspring livers. And the underlying mechanism regarding hepatic dysmetabolism caused by prenatal APAP exposure may be involved in disturbance of insulin-dependent AKT pathway (Wu et al., 2016). APAP administered intraperitoneally to mice (250 mg/kg) shows that mitochondrial GSH depletion appears to be more severe than cytoplasmic depletion (Tirmenstein and Nelson, 1989). Excessive APAP induces mitochondrial dysfunction through production of NAPQI binding to mitochondrial proteins, resulting in release of reactive oxygen species (ROS) to damage mitochondrial respiration and to affect ATP synthesis (Burcham and Harman, 1991; Ramsay et al., 1989; Jaeschke, 1990). The massive production of ROS in the liver is involved in APAP-induced hepatotoxicity *via* mediating endoplasmic reticulum (ER) stress (Uzi et al., 2013). In addition, high expression of peroxynitrite participation in the cytotoxicity of APAP may impair antioxidant function and cell homeostasis, gradually causing apoptotic or necrotic cell death in liver tissue (Denicola and Radi, 2005). Overall, current reports comprehensively uncover the molecular mechanism of APAP-induced hepatotoxicity, especially *via* mitochondrial avenue (Figure 4).

## 6 Conclusion

APAP exposure during pregnancy to treat pain or other symptoms may induce certain harmful effects on both the mother and the fetus. Other evidences indicate that prenatal APAP exposure may disrupt endocrine functions, including brain and liver tissues. Both intracellular and extracellular events are involved in pathophysiological processes in APAP-induced cytotoxicity, including drug metabolism, mitochondrial oxidative stress, DNA damage and microcirculatory dysfunction. As limited in current reference reports, more human data and toxicological investigation is needed to further elucidate the adverse actions of prenatal APAP exposure to offspring. More notably, we should pay close attention to the household use of APAP for safety, especially pregnant women before self-directed use.
